# Amyotrophic lateral sclerosis: an update on treatments from clinical trials

**DOI:** 10.1007/s00415-022-11553-6

**Published:** 2023-01-16

**Authors:** Ray Wynford-Thomas, Neil P. Robertson

**Affiliations:** 1 Department of Neurology, University Hospital of Wales, Heath Park, Cardiff, CF14 4XN UK; 2 Department of Neurology, Division of Psychological Medicine and Clinical Neuroscience, Cardiff University, University Hospital of Wales, Heath Park, Cardiff, CF14 4XN UK

## Introduction

Amyotrophic lateral sclerosis (ALS) is a progressive neurodegenerative disease resulting in loss of motor neurons, weakness, and death commonly due to respiratory failure, with a median survival of around 3–5 years. Despite considerable advances in the understanding of aetiology and pathophysiology, interventions that modify the natural disease course remain limited. At present available treatments can minimally prolong survival (riluzole) or slow functional decline (edaravone) and non-invasive ventilation (NIV) can prolong survival and improve quality of life in suitable patients, but tolerability can be a limiting factor. As a result, there remains a clear need for novel therapeutic approaches in ALS. In this month’s journal club, we review three clinical trials evaluating new treatments for ALS; tofersen, levosimendan and combined regulatory T-Lymphocyte/IL-2 therapy.

## Trial of antisense oligonucleotide tofersen for SOD1 ALS

Mutations in the gene encoding superoxide dismutase 1 (SOD1) result in destabilisation, disorder, and aggregation of the mutant SOD1 protein, and have been found to be associated with ALS. Tofersen is an antisense oligonucleotide which is administered intrathecally and reduces the synthesis of SOD1 protein.

In this phase 3 multi-centre trial, 108 participants were randomly assigned in a 2:1 ratio to receive a bolus of tofersen (100 mg; *n* = 72) or an equivalent volume of placebo (artificial cerebrospinal fluid; *n* = 36). Over 24 weeks participants received three doses once every 2 weeks, followed by five doses once every 4 weeks. The primary endpoint was a change in ALS Functional Rating Scale-Revised (ALSFRS-R) score from baseline to week 28, among participants predicted to have faster disease progression (*n* = 60), which was based upon SOD1 mutation type and the pre-study ALSFRS-R slope decline. No significant difference in change in ALSFRS-R score was found between the tofersen (*n* = 39) and placebo (*n* = 21) subgroups. Similarly, there were no differences observed in the secondary endpoints in this subgroup, which included slow vital capacity (SVC) and handheld dynamometry.

Ninety-five patients participated in an open-label extension and a subsequent combined analysis at week 52 found that all those who were given tofersen at the start of the trial, had a smaller decline in the ALSFRS-R score, percentage of predicted SVC, and handheld dynamometry megascore, compared to those who had delayed treatment. Furthermore, total SOD1 protein cerebrospinal fluid (CSF) concentrations and neurofilament light chain plasma concentrations, were also reduced in the early-start group.

Serious neurologic adverse events occurred in 7% of those who received tofersen and included myelitis, aseptic meningitis, lumbar radiculopathy, raised intracranial pressure and papilloedema. 58% of patients in the tofersen-group had a CSF pleocytosis.

*Comment*: Despite positive findings in biochemical profiles, this phase 3 trial reports no clinical benefit of treatment with tofersen in SOD1 ALS over 28 weeks. Although findings appear more positive at 52 weeks in those starting tofersen early, the authors comment that because there was no plan for adjustment of the widths of confidence intervals for multiple comparisons in the combined analysis, no conclusions can be drawn from these results. Further limitations included the need for imputation of 20% of the endpoint data and the results of the 28-week trial being known at the time of the analysis of the 52-week data. Given the risk of neurologic adverse events, treatment with tofersen is not without risk. In addition, the consequences of CSF pleocytosis in more than half of those treated, along with a raised CSF protein, should tofersen be used over longer periods, is unclear. Larger trials would be of value to further establish longer-term efficacy when initiated early in the disease course.

Miller et al. (2022) N Engl J Med. 387:1099–110.

## Safety and efficacy of oral levosimendan in people with amyotrophic lateral sclerosis (the REFALS study): a randomised, double-blind, placebo-controlled phase 3 trial

Levosimendan is a calcium sensitiser that increases myocyte contractility and has previously been used in decompensated cardiac failure due to its positive inotropic effect. In this multi-centre, phase 3 trial, 496 participants with laboratory-supported probable, probable, or definite ALS according to El Escorial revised criteria, were randomly allocated in a 2:1 ratio to oral levosimendan (*n* = 329) or placebo (*n* = 167). Participants had a disease duration of 12–48 months at entry (mean, 25.9 months) and required a sitting SVC between 60 and 90% of predicted normal. Patients taking riluzole (83%) or edaravone (18%) were included in the study. Participants were given 1 mg once a day for 2 weeks, increasing to 1 mg twice a day if tolerated, for 48 weeks.

There was no significant difference between treatment groups in the primary endpoint (change from baseline in supine SVC of predicted normal), nor did any secondary endpoints differ at 48 weeks (including change in the combined assessment of function and survival; a rank score based on the change in ALSFRS-R score adjusted according to survival time). Around a quarter of participants treated with levosimendan experienced adverse events leading to discontinuation, the most common being increased heart rate and headache. However, there was no difference in the percentage of serious adverse events (SAE’s) between groups. Fatal serious adverse events in the levosimendan-group (*n* = 27) included respiratory failure (*n* = 11), ALS (*n* = 4), respiratory depression (*n* = 3), and pulmonary embolism (*n* = 2).

*Comment*: Despite previously being shown to improve diaphragm neuromechanical efficiency by 21% in healthy volunteers, levosimendan did not significantly improve respiratory function or overall functionality in ALS. Furthermore, despite the lack of any new safety concerns, there was a relatively high discontinuation rate secondary to adverse events in the levosimendan-treated group. Limitations in the wider applicability of the study include inclusion criteria, which allowed for a relatively high disease duration at trial entry, and the lack of consideration of the pre-study disease progression rate. It may also be relevant to note that a high percentage of participants were also taking riluzole, which may have affected results. Future studies might be directed towards assessing the effect of higher doses of levosimendan in those with shorter disease duration and more rapidly progressive disease.

Cudkowicz et al. (2021) Lancet Neurol. 20: 821–31.

## Combined regulatory T lymphocyte and IL-2 treatment is safe, tolerable, and biologically active for 1 year in persons with amyotrophic lateral sclerosis

Regulatory T lymphocytes (Tregs) are a subpopulation of T cells (CD4^+^CD25^+^FOXP3^+^) that regulate immune system homeostasis, suppressing immune response, and thereby preventing autoimmunity. In ALS, normal Treg suppressive function is impaired and treatment with expanded autologous Tregs, alongside interleukin-2 (IL-2), has been proposed as a possible disease-modifying treatment.

In this 24-week randomised-controlled trial, 7 participants with possible, probable, laboratory-supported probable, or definite ALS based on El Escorial criteria, underwent leukapheresis and were then randomised in a 1:1 ratio to receive Treg infusions (1 × 106 cells/kg, intravenously every 4 weeks; *n* = 3) with IL-2 injections (2 × 105 IU/m^2^ injections 3 times per week) or placebo (*n* = 4). Mean disease duration was 17.7 months. Six participants entered a 24-week open-label extension, and 2 additional participants entered directly: all participants received increasing doses of Treg infusions/IL-2 injections (2 × 106 cells/kg and 3 × 106 cells/kg at 4-week intervals).

The primary endpoint (change in Treg suppressive function from baseline to week 24 assessed using peripheral blood) was not significantly different between groups. However, in those receiving Treg infusions, change in suppressive function was 26% higher. There was no evidence of a dose-dependent response in suppressive function or Treg numbers. Six out of 8 participants showed intermediate to no disease progression (as measured by the ALSFRS-R score) in a post-hoc analysis of the open-label extension. Although adverse events were common in Treg/IL-2 participants, they were generally mild and included injection site erythema, fatigue, myalgia, and headache. Several infections were also reported; urinary tract (*n* = 1), upper respiratory tract (*n* = 1), and COVID-19 (*n* = 1), and one participant had a raised alanine aminotransferase. However, no participants discontinued Treg/IL-2 therapy.

*Comment*: This study was limited by small participant numbers and larger trials are clearly needed to evaluate the efficacy and longer-term safety profile of Treg/IL-2 treatment in ALS. It should also be noted that at baseline 66% of participants in the RCT were on riluzole, 16% were on edaravone and 16% were using NIV, which may have affected results. The authors also comment that the use of Treg/IL-2 therapy may be limited in patients with elevated proinflammatory cytokines and oxidative stress markers, as found in the two participants who continued to rapidly progress despite treatment. Future trials therefore should consider the use of larger/more frequent doses to try and counter this, although dose-escalation in these 2 participants failed to result in a treatment response.

Thonhoff et al. (2022) Neurol Neuroimmunol Neuroinflamm. 9(6):e200019.

